# Fibroblast growth factor 23 and cognitive impairment: The health, aging, and body composition study

**DOI:** 10.1371/journal.pone.0243872

**Published:** 2020-12-11

**Authors:** David A. Drew, Ronit Katz, Stephen Kritchevsky, Joachim H. Ix, Michael Shlipak, Anne B. Newman, Andy Hoofnagle, Linda Fried, Orlando M. Gutiérrez, Mark Sarnak

**Affiliations:** 1 Tufts Medical Center, Boston, MA, United States of America; 2 University of Washington, Seattle, WA, United States of America; 3 Wake Forest School of Medicine, Winston-Salem, NC, United States of America; 4 University of California San Diego School of Medicine, San Diego, CA, United States of America; 5 Kidney Health Research Collaborative, San Francisco VA Health Care System and University of California San Francisco, San Francisco, CA, United States of America; 6 University of Pittsburgh Graduate School of Public Health, PA, United States of America; 7 VA Pittsburgh Healthcare System, Pittsburgh PA and University of Pittsburgh School of Medicine, Pittsburgh, PA, United States of America; 8 University of Alabama at Birmingham, Birmingham, AL, United States of America; Nathan S Kline Institute, UNITED STATES

## Abstract

**Background:**

Concentrations of fibroblast growth factor 23 (FGF-23), a hormone that regulates phosphorus and vitamin D metabolism, increase as kidney function declines. Excess fibroblast growth factor 23 may impact brain function through promotion of vascular disease or through direct effects on neuronal tissue.

**Methods:**

In the Healthy Aging and Body Composition Study, a longitudinal observational cohort of well-functioning older adults, intact serum FGF-23 was assayed in 2,738 individuals. Cognitive function was assessed at baseline and longitudinally at years 3, 5, and 8 by administration of the Modified Mini Mental State Examination (3MSE), a test of global cognitive function, and the Digit Symbol Substitution Test (DSST), a test primarily of executive function. The associations between FGF-23 and baseline cognitive function and incident cognitive impairment were evaluated using logistic and Poisson regression respectively, and were adjusted for demographics, baseline estimated glomerular filtration rate (eGFR), urine albumin/creatinine ratio, comorbidity, and other measures of mineral metabolism including soluble klotho.

**Results:**

The mean (SD) age was 74(3) years, with 51% female, and 39% black. The median (25th, 75th) FGF-23 concentration was 47 pg/mL (37, 60). Three hundred ninety-two individuals had prevalent cognitive impairment by the 3MSE and 461 by the DSST. There was no observed association between FGF-23 and baseline cognitive function for either cognitive test. There were 277 persons with incident cognitive impairment by 3MSE, and 333 persons with incident cognitive impairment by DSST. In fully adjusted models, each two-fold higher concentration of baseline FGF-23 was not associated with incident cognitive impairment by the 3MSE (IRR = 1.02[0.88, 1.19] fully adjusted model) or by the DSST (IRR = 0.98 [0.84, 1.15]. We saw no difference when analyses were stratified by eGFR greater than or less than 60 ml/min/1.73m^2^.

**Conclusion:**

Intact FGF-23 was not associated with baseline cognitive function or incident cognitive impairment in this cohort well-functioning older adults.

## Introduction

Older adults are at high risk of developing both cognitive impairment and chronic kidney disease (CKD). Accordingly, cognitive impairment is highly prevalent in patients with CKD and increases in both prevalence and severity in older individuals with more advanced stages of kidney disease [1]. Although traditional vascular risk factors such as hypertension, hyperglycemia and dyslipidemia are likely important risk factors for both conditions, they do not fully explain the high incidence and severity of cognitive impairment in CKD [2].

Fibroblast growth factor 23 (FGF-23) is a circulating hormone that is involved in the regulation of phosphorus balance and vitamin D. Concentrations of FGF-23 increase as eGFR declines, and the rise in FGF-23 is associated with increased risk for cardiovascular events and mortality [3]. FGF-23 is also expressed within the brain [4] and increased concentrations of FGF-23 have been associated with higher rates of stroke [3]. In a cross-sectional study of maintenance hemodialysis patients, higher FGF-23 levels were independently associated with worse memory function [5]. Several studies have also examined the association of FGF-23 with either dementia or cognitive impairment in patient cohorts without significant kidney disease; two studies found no association and the remaining study demonstrated a relationship with incident dementia but not with cognitive decline [6–8]. As increases in FGF-23 appear primarily driven by decreased kidney function, there remains uncertainty about FGF-23’s potential role in the development of cognitive impairment in groups at higher risk for both cognitive impairment and kidney disease, such as older adults.

Given the potential for FGF-23 to impact the brain, we hypothesized that high FGF-23 concentrations would be associated with the development of cognitive impairment. We specifically chose to examine this relationship in the Health Aging and Body Composition Study (Health ABC), a diverse cohort of well-functioning older adults, due to the high prevalence and risk of cognitive impairment in this population. This cohort also has existing measures of kidney function and other mineral metabolism markers such as calcium, phosphorus, parathyroid hormone (PTH), vitamin D 25OH, and soluble klotho, allowing for adjustment of these parameters. We therefore evaluated the independent association of intact FGF-23 with baseline cognitive function and incident cognitive impairment.

## Methods

### Study population

Health ABC is a prospective cohort initiated in 1997 with a goal of assessing how weight-related health conditions impact age-related physiologic and functional status. The study population consists of 3,075 participants aged 70–79 years at baseline with equal numbers of men and women and 38% black. All persons included were determined to be free of disability in activities of daily living and free of mobility limitation at baseline. All participants who had measures of FGF-23 at year two (one year after baseline) and cognitive test scores at baseline (n = 2,738) were included in the current study. Samples from this time point were chosen because measures of calcium, phosphorus, PTH, vitamin D 25(OH) and soluble klotho were also available at year 2. The Cognitive Vitality Sub-study (n = 929) is a subset of the Health ABC cohort initiated in year 3 in which a more detailed cognitive battery was administered in years 3, 5, 7, and 9 which included four additional cognitive tests. All participants provided written consent for the study. The Health ABC study was approved by institutional review boards at the Universities of Pittsburgh and Tennessee and meets the requirements of the declaration of Helsinki.

### Exposure

FGF-23 was measured using a commercial ELISA that detects the full-length intact peptide (*Kainos Laboratories*, *Japan)*. Serum samples from the year 2 visit (1998–1999) were stored at -70°C and were not thawed until time of analysis (2015). This FGF-23 assay has a limit of detection of 3 pg/mL. Samples were assayed in batches over two months and the between-batch coefficient of variation was 10.7%. A subset of samples were blindly measured in duplicate and displayed an intra-sample coefficient of variation of 3.1%.

### Outcomes

#### Baseline cognitive test scores

The modified mini-mental status examination (3MSE) measures components of orientation, concentration, language, praxis, and immediate and delayed memory [9]. The Digit Symbol Substitution test (DSST) measures attention and executive function [10]. Secondary outcomes include tests from the Cognitive Vitality sub-study: Buschke Selective Reminding test [11] (verbal memory), Boxes and Digit Copying tests [12] (psychomotor speed), 15 item Executive interview [13] (executive function), and the Pattern and Letter Comparison tests [12] (attention and perceptual speed).

### Longitudinal

#### Incident cognitive impairment

The 3MSE was administered at baseline, as well as years 3, 5 and 8, while the DSST was administered at baseline and years 5 and 8. For the 3MSE, incident cognitive impairment was defined as a score of less than 80 at any point during follow up and at least a one standard deviation decline in baseline score (to limit inclusion of individuals with baseline scores modestly above 80 who subsequently decline below 80), as has been defined in other studies [14,15]. Participants with a score less than 80 at first visit were excluded from this analysis. For the DSST, incident cognitive impairment was defined as a score decline of ≥ 1 standard deviation from mean baseline score at any point during follow up [16].

### Covariates

All covariates were obtained at study enrollment, with the exception of measures of mineral metabolism, which were assayed in samples obtained from the year two visit. Cardiovascular disease (CVD) status was defined as a prior history of coronary artery disease, stroke, or heart failure. Diabetes was defined as use of hypoglycemic agents, self-reported history, fasting plasma glucose level ≥ 126 mg/dL, or 2-hour oral glucose tolerance test result ≥ 200 mg/dL. Systolic and diastolic blood pressures were obtained from the right arm by trained and certified clinical staff using a conventional mercury sphygmomanometer with the participant in a seated position. Hypertension was defined as self-reported physician's diagnosis of hypertension, confirmed by current use of antihypertensive medications.

Cystatin C was measured from baseline samples at the Health ABC core laboratory (University of Vermont, Burlington, VT) using a BNII nephelometer (Dade Behring Inc., Deerfield, IL), a particle-enhanced immunonepholometric assay (N Latex Cystatin C) [17]. Estimated glomerular filtration rate (eGFR) was calculated using a validated cystatin C based estimating equation [18,19]. Urine albumin to creatinine ratio (UACR) was measured from stored samples from the baseline visit. Urine albumin was measured using a particle-enhanced turbidimetric inhibition immunoassay allowing for direct albumin quantification (Siemens), while urine creatinine was measured by a modified Jaffé method on a clinical chemistry analyzer (Siemens). Measures of mineral metabolism including calcium, phosphorus, and PTH were measured at year two, concurrent with FGF-23 measurement, from frozen stored samples. Intact PTH was measured in EDTA plasma using a two-site immunoradiometric assay kit (N-tact PTHSP; DiaSorin). Serum calcium and phosphorus levels were measured using direct quantitative colorimetric determination (Stanbio Laboratory, Boerne, TX, USA). Serum 25(OH) Vitamin D was measured using a two-step radioimmunoassay (25-Hydroxyvitamin D 125I RIA Kit, DiaSorin, Stillwater, MN, USA). Soluble klotho was assayed using a commercially available sandwich ELISA test (IBL-International, Japan) from never thawed frozen serum stored at -70°C obtained at the year two visit. This assay is reported to have a sensitivity of 6.15 pg/mL [20], and demonstrated an inter-assay coefficient of 18%.

### Statistical analysis

We examined baseline characteristics of participants across quartiles of FGF-23. These were summarized with means and standard deviations, or medians and interquartile ranges (IQR) for highly skewed variables or proportions for categorical variables. For eGFR and UACR, we also included the proportion of participants in each quartile below or above clinically relevant cut points (eGFR < 60 ml/min/1.73m^2^, UACR > 30 mg/g).

### FGF-23 and baseline cognitive function

Multivariable linear regression models were used to assess the relationship between FGF-23 with the absolute score on the 3MSE and DSST. Multivariable logistic regression was used to determine the association between FGF-23 with a score of 3MSE < 80, and FGF-23 with a score on the DSST of less than 1 SD below the baseline mean. FGF-23 was examined as a continuous variable (log base 2, so that the interpretation would be “two-fold higher” of the exposure) and categorized by quartiles. Model 1 was unadjusted. Multivariable models were then sequentially constructed through a series of nested models using pre-specified variables as follows: Model 2: adjusted for age, sex, race, study site and education; Model 3: additionally adjusted for baseline eGFR, diabetes, prevalent CVD, hypertension, and urine ACR; Model 4: additionally adjusted for calcium, phosphorus, PTH, 25(OH) Vitamin DH and soluble klotho. Based on *a priori* hypotheses that the relationship between FGF-23 and cognitive function could be modified by eGFR, we performed stratified analyses in which the above models were repeated for those with baseline eGFR ≥60 ml/min/1.73m^2^ and those with eGFR < 60 ml/min1.73m^2^.

### FGF-23 and incident cognitive impairment

Poisson (log-link) regression was used to model the incidence rate ratio of cognitive impairment as a function of FGF-23 concentration with robust variance estimation and an offset for follow-up time. Exposure variables were again examined as both linear terms and by category (quartiles) to assess for non-linear relationships based on our spline analysis. Identical multivariable models were constructed as described above, including interaction terms.

### Secondary analyses

For the participants in the Cognitive vitality sub-study, we examined the association of FGF-23 with cognitive test scores using linear regression with identical multivariable models to above. Specifically, we carried the year 2 FGF-23 value forward to year 3, and conducted analyses as though they were in true cross-section, as these measures were only available at visits one year apart. We also explored the association between FGF-23 and change in cognitive test scores over time using linear mixed models with the same model structure.

Analyses were conducted using SPSS (IBM Corp. Released 2015. IBM SPSS Statistics for Windows, Version 23.0. Armonk, NY: IBM Corp.) and Stata (StataCorp. 2013. *Stata Statistical Software*: *Release 13*. College Station, TX: StataCorp LP.) A two sided p value of < 0.05 was considered statistically significant for all analyses including interaction terms.

## Results

### Baseline characteristics

Among 3,075 persons, 300 participants did not have sample available for measuring FGF-23, while 37 participants were missing cognitive test scores at baseline, leaving 2,738 with complete data available for analysis. For longitudinal analyses, 114 participants were missing 3MSE scores while 278 had a 3MSE score of less than 80 at baseline, leaving 2,346 available for analysis. For the DSST, 461 were missing scores on follow up, leaving 2,277 available for longitudinal analysis. The median FGF-23 level was 46 pg/mL (37–60 (25^th^– 75^th^ percentile)). The average (SD) age was 74 (3) years, with 51% female, and 39% black (Table 1). The mean baseline eGFR was 72 (18) ml/min/1.73m^2^ and 25% of participants had a baseline eGFR of less than 60 ml/min/1.73m^2^.

**Table 1:** Demographics and clinical characteristics by quartiles of FGF-23.

Variable	Full cohort	Quartile 1	Quartile 2	Quartile 3	Quartile 4
Range		1.41–36.68	36.69–46.65	46.66–60.16	60.17–1615.79
N	2738	683	685	686	684
Age (years)	74 (3)	73 (3)	74 (3)	74 (3)	74 (3)
Female	1403 (51%)	381 (56%)	357 (52%)	322 (47%)	343 (50%)
Black	1073 (39%)	295 (43%)	253 (37%)	253 (37%)	272 (40%)
Education					
< HS	643 (24%)	173 (25%)	151 (22%)	156 (23%)	163 (24%)
HS grad	884 (32%)	226 (33%)	230 (34%)	217 (32%)	211 (31%)
postsecondary	1204 (44%)	283 (42%)	301 (44%)	313 (46%)	307 (45%)
Diabetes	979 (36%)	211 (31%)	239 (35%)	253 (37%)	276 (40%)
Hypertension	2017 (74%)	472 (69%)	476 (70%)	521 (76%)	548 (80%)
HTN meds use	1489 (55%)	297 (44%)	343 (50%)	389 (57%)	460 (68%)
Coronary Artery Disease	492 (18%)	93 (14%)	108 (16%)	131 (19%)	160 (24%)
Heart Failure	32 (1.2%)	3 (0.4%)	3 (0.4%)	3 (0.4%)	23 (3.5%)
Cerebrovascular Disease	190 (7%)	52 (8%)	36 (5%)	47 (7%)	55 (8%)
Systolic Blood Pressure (mmHg)	135 (21)	134 (20)	135 (20)	136 (21)	138 (22)
Diastolic Blood Pressure (mmHg)	71 (12)	70 (12)	72 (11)	71 (12)	72 (12)
Body Mass Index (kg/m2)	27.3 (4.7)	26.7 (4.7)	27.0 (4.7)	27.4 (4.5)	28.1 (4.9)
Total Cholesterol (mg/dL)	203 (39)	202 (38)	204 (39)	201 (37)	203 (41)
LDL (mg/dL)	122 (35)	120 (35)	123 (34)	121 (34)	123 (36)
HDL (mg/dL)	54 (17)	57 (18)	55 (18)	52 (16)	51 (15)
Statin use	349 (13%)	73 (11%)	78 (11%)	107 (16%)	91 (13%)
C-reactive protein (mg/L)	1.65 [0.99, 3.07]	1.61 [0.96, 3.01]	1.48 [0.92, 2.75]	1.63 [1.00, 3.10]	1.98 [1.09, 3.48]
eGFR_cysC (ml/min/1.73m2)	72 (18)	77 (17)	77 (17)	72 (17)	64 (20)
eGFR_cysC < 60	670 (25%)	104 (15%)	120 (18%)	168 (25%)	278 (41%)
Urine Albumin/Creatinine ratio (mg/g)	7.59 [3.92, 18.97]	7.18 [3.66, 15.76]	7.02 [3.81, 16.16]	7.27 [3.37, 17.12]	9.56 [4.78, 31.30]
UACR ≥ 30	471 (18%)	89 (13%)	96 (14%)	115 (17%)	171 (26%)
Calcium (mg/dL)	8.87 (0.43)	8.80 (0.39)	8.84 (0.41)	8.89 (0.44)	8.95 (0.47)
Phosphorus (mg/dL)	3.55 (0.48)	3.51 (0.44)	3.52 (0.47)	3.53 (0.48)	3.64 (0.51)
Parathyroid hormone (pg/ml)	33.6 [22.1, 45.7]	31.7 [23.5, 41.7]	31.2 [24.1, 41.4]	34.1 [25.7, 45.3]	38.1 [27.7, 55.9]
25(OH) vitD (ng/ml)	26 (11)	24 (12)	26 (10)	27 (11)	27 (12)
Soluble klotho (pg/mL)	683.1 (325.7)	710.0 (352.2)	700.8 (354.4)	679.6 (307.1)	642.0 (279.3)

Across quartiles of FGF-23, those in the highest quartile were more likely to have comorbid conditions such as diabetes, hypertension, coronary artery disease, and heart failure. In addition, those in the highest quartile also had lower baseline eGFR, were more likely to have a spot urine ACR > 30 mg/g, had higher serum calcium, phosphorus, 25(OH) Vitamin D and PTH concentrations, and had lower soluble klotho concentrations. There were no observed differences in age, sex, and black race by category of FGF-23.

### FGF-23 and baseline cognitive function

Two hundred seventy-eight participants had a baseline 3MSE score < 80, classifying them as cognitively impaired. Higher FGF-23 concentrations were not associated with 3MSE scores or with 3MSE score < 80 at baseline (Table 2). This finding was unchanged throughout all multivariable models, including after adjustment for demographics, baseline kidney function, CKD and CVD risk factors, and other measures of mineral metabolism. Similarly, higher FGF-23 was not associated with DSST scores across all models.

**Table 2:** Association of FGF-23 with baseline cognitive function in the health ABC study.

FGF-23	N	N with outcome	Model 1	Model 2	Model 3
		
**3MSE score**			β (95% CI)	β (95% CI)	β (95% CI)
Continuous (per 2-fold higher)	2738	-	-0.20 (-0.63, 0.24)	-0.10 (-0.49, 0.28)	-0.13 (-0.52, 0.27)
Quartiles					
< 36	683	-	0 (ref)	0 (ref)	0 (ref)
36–46	685	-	0.76 (-0.08, 1.60)	0.30 (-0.40, 0.99)	0.29 (-0.41, 0.98)
47–60	686	-	0.43 (-0.40, 1.27)	0.27 (-0.43, 0.97)	0.26 (-0.44, 0.97)
> 60	684	-	-0.12 (-0.96, 0.72)	0.01 (-0.72, 0.73)	-0.02 (-0.76, 0.73)
**3MSE < 80 at baseline**			OR (95% CI)	OR (95% CI)	OR (95% CI)
Continuous (per 2-fold higher)	2738	278	1.09 (0.91, 1.31)	1.05 (0.86, 1.29)	1.10 (0.89, 1.37)
Quartiles					
< 36	683	79 (12%)	1.00 (ref)	1.00 (ref)	1.00 (ref)
36–46	685	61 (9%)	0.71 (0.49, 1.01)	0.78 (0.52, 1.16)	0.79 (0.53, 1.18)
47–60	686	62 (9%)	0.77 (0.54, 1.09)	0.80 (0.54, 1.18)	0.82 (0.55, 1.21)
> 60	684	76 (11%)	0.93 (0.66, 1.30)	0.84 (0.56, 1.24)	0.88 (0.58, 1.33)
**DSST score**			β (95% CI)	β (95% CI)	β (95% CI)
Continuous (per 2-fold higher)	2738	-	0.04 (-0.77, 0.86)	0.21 (-0.45, 0.87)	0.24 (-0.45, 0.92)
FGF-23 Quartiles					
< 36	683	-	0 (ref)	0 (ref)	0 (ref)
36–46	685	-	0.58 (-0.98, 2.13)	-0.32 (-1.52, 0.89)	-0.33 (-1.54, 0.88)
47–60	686	-	0.28 (-1.27, 1.84)	0.06 (-1.15, 1.27)	0.04 (-1.18, 1.27)
> 60	684	-	0.17 (-1.43, 1.70)	0.48 (-0.76, 1.75)	0.56 (-0.73, 1.85)

Model 1 = unadjusted analysis.

Model 2 = adjusted for age, sex, race, study site, and education, diabetes, cardiovascular disease, hypertension, eGFR and ACR.

Model 3 = M2 + calcium, phosphorus, PTH, 25(OH) Vitamin D and klotho.

### FGF-23 and incident cognitive impairment

The mean (SD) follow up time was 5.8 years (1.9). Three hundred forty-one participants developed cognitive impairment by the 3MSE, while 333 developed cognitive impairment by the DSST over 8 years of follow up (Table 3). Each two-fold higher FGF-23 was not associated with a higher incident rate ratio for cognitive impairment by the 3MSE, and was unchanged after adjustment for demographics, comorbidity, and other measures of mineral metabolism. Across quartiles of FGF-23, those in the highest quartile compared to the lowest quartile were the most likely to develop cognitive impairment, though this result was not statistically significant. Similarly, no associations were not seen with incident cognitive impairment defined by the DSST, across all models.

**Table 3:** Association of FGF-23 with incident cognitive impairment in the health ABC study.

FGF-23	N	N with outcome	Model 1	Model 2	Model 3
			IRR (95% CI)	IRR (95% CI)	IRR (95% CI)
**3MSE < 80**					
Continuous (per 2-fold higher)	2346	341	1.08 (0.91, 1.29)	1.08 (0.93, 1.27)	1.02 (0.88, 1.19)
Quartiles					
< 36	590	86	1.00 (ref)	1.00 (ref)	1.00 (ref)
36–46	596	73	0.93 (0.66, 1.32)	1.01 (0.71, 1.44)	0.96 (0.67, 1.37)
47–60	594	86	1.17 (0.85, 1.62)	1.28 (0.93, 1.77)	1.22 (0.86, 1.69)
> 60	566	96	1.37 (1.00, 1.89)	1.34 (0.97, 1.87)	1.23 (0.88, 1.71)
**Incident DSST score < 1SD**			IRR (95% CI)	IRR (95% CI)	IRR (95% CI)
Continuous (per 2-fold higher)	2277	333	0.98 (0.83, 1.12)	0.95 (0.82, 1.11)	0.95 (0.82, 1.11)
FGF-23 Quartiles					
< 36	591	82	1.00 (ref)	1.00 (ref)	1.00 (ref)
36–46	570	97	1.23 (0.93, 1.62)	1.21 (0.91, 1.62)	1.18 (0.88, 1.57)
47–60	580	74	0.90 (0.67, 1.22)	0.89 (0.66, 1.21)	0.88 (0.65, 1.20)
> 60	536	80	1.08 (0.81, 1.45)	1.04 (0.77, 1.40)	1.04 (0.76, 1.43)

Model 1 = unadjusted analysis.

Model 2 = adjusted for age, sex, race, study site, education, diabetes, cardiovascular disease, hypertension, eGFR and urine ACR.

Model 3 = M2 + calcium, phosphorus, PTH, 25(OH) Vitamin D and klotho.

### Stratified analyses

When analyses were stratified by eGFR (≥60 ml/min/1.73m^2^ vs < 60 ml/min/1.73m^2^), the association between FGF-23 and each cognitive outcome was unchanged. We have included the results for association between FGF-23 and incident 3MSE < 80 and incident DSST < 1SD in S1 Table.

#### Secondary analyses of the cognitive vitality sub-study

Among 929 participants in the Cognitive Vitality Study, 843 individuals had both samples available for FGF-23 and complete cognitive testing available. There were no associations between FGF-23 and baseline cognitive tests scores including with Verbal memory, Psychomotor speed, Perceptual speed, and Executive function (Table 4). In longitudinal analyses, there were similarly no associations between FGF-23 and annual change in scores of each of the cognitive tests (Table 5).

**Table 4:** Association of FGF-23 with baseline cognitive vitality test scores.

N = 843 participants	Model 1	Model 2	Model 3
Outcome*	β (95% CI)	β (95% CI)	β (95% CI)
3MS Score	-0.001 (-0.014, 0.012)	-0.002 (-0.012, 0.009)	-0.002 (-0.013, 0.009)
DST score	-0.004 (-0.053, 0.044)	-0.015 (-0.061, 0.031)	-0.013 (-0.060, 0.034)
Verbal Memory			
Immediate Recall	-0.011 (-0.053, 0.032)	-0.005 (-0.043, 0.033)	-0.008 (-0.047, 0.031)
Delayed recall	-0.015 (-0.11, 0.08)	0.003 (-0.086, 0.093)	0.003 (-0.089, 0.095)
Psychomotor Speed	0.018 (-0.025, 0.060)	0.018 (-0.018, 0.054)	0.027 (-0.01, 0.063)
Perceptual Speed	-0.006 (-0.060, 0.047)	-0.001 (-0.046, 0.044)	0.003 (-0.043, 0.049)
Depression	-0.011 (-0.0140, 0.118)	-0.032 (-0.163, 0.098)	-0.048 (-0.182, 0.085)
Executive Function			
EXIT15	-0.036 (-0.134, 0.061)	-0.039 (-0.123, 0.046)	-0.05 (-0.136, 0.037)
CLOX1	-0.020 (-0.055, 0.015)	-0.021 (-0.056, 0.014)	-0.02 (-0.056, 0.016)

Model 1 = unadjusted analysis.

Model 2 = adjusted for age, sex, race, study site, education, diabetes, cardiovascular disease, hypertension, eGFR and urine ACR.

Model 3 = M2 + calcium, phosphorus, PTH, 25(OH) Vitamin D and klotho.

*All dependent variables were log transformed.

** FGF-23 was log transformed.

**Table 5:** Association of FGF-23 with annualized relative change in cognitive vitality test scores.

N = 843 participants	Model 1	Model 2	Model 3
Outcome*	β (95% CI)	β (95% CI)	β (95% CI)
3MS Score	0.07 (-0.17, 0.31)	0.03 (-0.21, 0.26)	0.03 (-0.21, 0.26)
DST score	-0.04 (-1.56, 1.48)	0.06 (-1.43, 1.54)	0.09 (-1.39, 1.58)
Verbal Memory			
Immediate Recall	-0.15 (-1.96, 1.67)	-0.58 (-2.33, 1.17)	-0.55 (-2.33, 1.19)
Delayed recall	-0.42 (-1.28, 0.44)	-0.56 (-1.43, 0.31)	-0.54 (-1.42, 0.33)
Psychomotor Speed	0.42 (-0.26, 1.09)	0.33 (-0.33, 0.98)	0.34 (-0.33, 0.98)
Perceptual Speed	0.47 (-0.50, 1.45)	0.33 (-0.62, 1.28)	0.33 (-0.62, 1.28)
Depression	0.62 (-2.08, 3.32)	0.45 (-2.24, 3.13)	0.42 (-2.26, 3.11)
Executive Function			
CLOX1	-0.46 (-1.22, 0.31)	-0.32 (-1.07, 0.42)	-0.32 (-1.07, 0.43)

Model 1 = unadjusted analysis.

Model 2 = adjusted for age, sex, race, study site, education, diabetes, cardiovascular disease, hypertension, eGFR and urine ACR.

Model 3 = M2 + calcium, phosphorus, PTH, 25(OH) Vitamin D and klotho.

*All dependent variables were log transformed.

** FGF-23 was log transformed.

*** Coefficient interpretation example 3MSE & FGF-23: Every two fold higher FGF-23 is associated with a 0.07% decline in 3MSE score.

## Discussion

In a diverse cohort of older adults, we found no association between intact FGF-23 and baseline cognitive function or with incident cognitive impairment as assessed by two distinct cognitive tests. Overall, we conclude that there is unlikely to be a relationship between FGF-23 and cognitive function and that previous reported associations may have been confounded by other risk factors for cognitive impairment, such as kidney function (eGFR and albuminuria) and other measures of mineral metabolism including 25(OH) Vitamin D.

There are limited published data on the association of FGF-23 with cognitive impairment. A study of 263 participants on maintenance hemodialysis demonstrated a cross-sectional association between higher c-terminal FGF-23 and worse baseline memory function, but not executive function [21]. The outcomes were based on composite measures of memory and executive function each representing several cognitive tests and the relationships persisted after adjustment for demographics, comorbid conditions, and measures of mineral metabolism. Limitations of that study are that it was cross-sectional and represented a select population with. kidney failure requiring dialysis. The Homocysteine Study Cognitive Function Substudy, which included 600 participants with advanced CKD as well as participants requiring maintenance hemodialysis, found no cross-sectional association between c-terminal FGF-23 and cognitive impairment, though the cognitive outcome was based on a single telephone based cognitive test [8]. The Reasons for Geographic and Racial Differences in Stroke (REGARDS) study measured c-terminal FGF-23 and found no association with incident cognitive impairment using a case-control design [7]. Finally, McGrath et al. recently published an analysis of the Framingham Offspring Study which examined the association of c-terminal FGF-23 with structural brain disease, cognitive performance, and incident dementia within 1,563 community dwelling adults [6]. Higher FGF-23 was associated with incident dementia, but not with cognitive test score slopes or with structural brain disease. Critically, this relationship was attenuated and became non-significant in the final statistical model which adjusted for eGFR and 25(OH) vitamin D.

There are several potential mechanisms which may explain observed associations between FGF-23 and cognitive impairment. First, FGF-23 may simply be a sensitive biomarker of impaired kidney function or clearance as shown by its rise prior to other metabolic abnormalities seen in CKD [22,23]. Kidney disease is strongly associated with cognitive impairment and therefore the observed association of FGF-23 and cognitive impairment may represent residual confounding with kidney function, kidney disease severity. Second, the association may be related to FGF-23’s known physiologic mechanisms of action. Elevations in FGF-23 appear to occur in response to states of phosphorus excess such as CKD or high phosphorus intake, suggesting that FGF-23 may be a sensitive marker of overall phosphorus burden [24]. Since excess phosphorus is thought to be a potential promoter of dystrophic calcification, FGF-23 concentrations may themselves lead to cerebrovascular disease states such as white matter disease, subclinical stroke, and clinical stroke. Supporting this possibility, an analysis of the same Northern Manhattan Study cited previously found that higher FGF-23 levels were associated with a greater incidence of white matter disease and subclinical infarcts as seen on brain magnetic resonance imaging [25]. Alternatively, this finding could also be explained by FGF-23’s potential impact on the heart to promote left ventricular hypertrophy [26,27], which in turn may increase the risk of stroke [28,29] and cerebrovascular induced cognitive impairment [30].

The strengths of this study include a large and diverse cohort, detailed ascertainment of risk factors and repeated measures of cognitive function over a moderate follow up time period. We utilized an assay which measures only the biologically active intact FGF-23; use of the c-terminal assay may yield FGF-23 levels including fragments that are increased in states of inflammation, a potential confounder for cognitive impairment [31]. We also have more detailed cognitive testing in a subset of the Health ABC participants, allowing for additional assessment of possible associations. There are several limitations to the current study. We did not have simultaneous assessment of the exposure (FGF-23 assayed at year 2) and year 1 covariates, including kidney function, though the measures were within one year of each other, and it is not apparent if within subject changes in FGF-23 over time [32] impact associations with clinical outcomes. Fibroblast growth factor 23 was also measured in sixteen to seventeen year old store samples. Though these samples were never thawed and stored at -80C, there remains the possibly that degradation of the FGF-23 protein occurred over this prolonged period of time. A prior study showed that such degradation was detectable and resulted in a consistent but slight reduction in measured FGF-23 [33]. It is therefore possible that over time, degradation in FGF-23 could result in a small bias towards to the null, leading to the lack of an observed association. Additionally, our cohort did not include any participants with advanced CKD at baseline, so we are unable to generalize our results to group with substantially higher baseline FGF-23 concentrations. Finally, we did not have detailed cognitive measures available in all participants.

In summary, we found no between FGF-23 and either cross-sectional cognitive function or longitudinal cognitive impairment. These results strongly suggest that FGF-23 is unlikely to be a major contributor to cognitive impairment in well-function older adults. Further study is needed to determine if these results apply to populations with higher FGF-23 levels, such as those with advanced CKD or with kidney failure requiring maintenance hemodialysis.

## Supporting information

S1 Table Association of FGF-23 and 3MSE<80 and change in DSST stratified by CKD status. (DOCX)
