# Supplementary material for: Fibroblast growth factor 23 and cognitive impairment: The health, aging, and body composition study
Source: PLoS One. 2020 Dec 11;15(12):e0243872. doi: 10.1371/journal.pone.0243872 (PMC7732072; doi:10.1371/journal.pone.0243872)
Supplement: S1 Table — (DOCX) [file pone.0243872.s001.docx]

| **Supplemental Table 1. Association of FGF-23 and 3MSE<80 and change in DSST stratified by CKD status** | | | | | | |
| --- | --- | --- | --- | --- | --- | --- |
| **Two-fold higher FGF-23** | |  |  | **Model 1** | **Model 2** | **Model 3** |
|  |  | **N** | **N with outcome** | OR (95% CI) | OR (95% CI) | OR (95% CI) |
| **Incident 3MSE < 80** | **eGFR ≥ 60** | 2045 | 201 | 1.14 (0.90, 1.46) | 1.16 (0.91, 1.47) | 1.11 (0.87, 1.42) |
|  | **eGFR < 60** | 669 | 75 | 0.93 (0.69, 1.25) | 0.93 (0.77, 1.11) | 0.89 (0.73, 1.09) |
|  |  | N |  | β (95% CI) | β (95% CI) | β (95% CI) |
| **Change in DSST score** | **eGFR ≥ 60** | 2045 | - | 0.16 (-0.95, 1.26) | 0.02 (-0.83, 0.86) | 0.08 (-0.77, 0.93) |
|  | **eGFR < 60** | 669 | - | 0.30 (-0.97, 1.57) | 0.12 (-0.91, 1.14) | 0.43 (-0.62, 1.48) |
| Model 1 = unadjusted analysis.  Model 2 = adjusted for age, sex, race, study site, education, diabetes, cardiovascular disease, hypertension, eGFR and urine ACR.  Model 3 = M2 + calcium, phosphorus, PTH, 25(OH) Vitamin D and klotho  *All dependent variables were log transformed | | | | | | |
